# Exploring the incidence of dysplasia or adenocarcinoma in early onset Barrett’s esophagus

**DOI:** 10.1055/a-2386-7843

**Published:** 2024-09-23

**Authors:** Irma C. Noordzij, Clément J. Huysentruyt, Wouter L. Curvers, Gesina van Lijnschoten, Ad A. M. Masclee, Erik J. Schoon

**Affiliations:** 1 3168 Gastroenterology and Hepatology, Catharina Ziekenhuis, Eindhoven, Netherlands; 2 5211 GROW School for Oncology and Reproduction, Maastricht University, Maastricht, Netherlands; 3 159178 Pathology, Eurofins PAMM BV Laboratorium voor Pathologie, Eindhoven, Netherlands; 4 199236 Gastroenterology and Hepatology, Maastricht University Medical Centre+, Maastricht, Netherlands; 5 568601 Onocology, Maastricht University GROW School for Oncology and Reproduction, Maastricht, Netherlands

## Abstract

**Background** Currently data on the risk of progression to and lifetime risk of cancer are not available for patients with young onset Barrett’s esophagus (BE). Our aim was to obtain epidemiologic data on the incidence of dysplasia or adenocarcinoma in young onset BE in the Netherlands by collecting data on all histologically confirmed cases over a prolonged period of 25 years between January 1, 1991 and December 31, 2015.

**Methods** Data were obtained from the Dutch National Pathology Registry. Patients were included if there was a suspicion of BE visualized in the esophagus during the endoscopic examination in combination with a concordant histologic diagnosis of intestinal metaplasia.

**Results** 231 patients with early onset BE were identified (median age 26 years [range 0–29 years]), with 17 progressing to dysplasia (6 prevalent and 11 incident). For the patients with incident dysplasia, the median surveillance time between the diagnosis of early onset BE and diagnosis of dysplasia was 5 years (range 0–16 years). The incidence rate of dysplasia was 7.3 per 1000 person-years. There were three patients who developed adenocarcinoma (1 prevalent and 2 incident), who were diagnosed at ages 28, 35, and 36 years. The incidence rate of adenocarcinoma was 1.3 per 1000 person-years.

**Conclusions** In this 25-year period, 231 patients were diagnosed with early onset BE in the Netherlands, with 17 patients progressing to dysplasia and three developing adenocarcinoma. This corresponded to incidence rates of 7.3 per 1000 person-years for dysplasia and 1.3 per 1000 person-years for adenocarcinoma.

## Abstract (de)

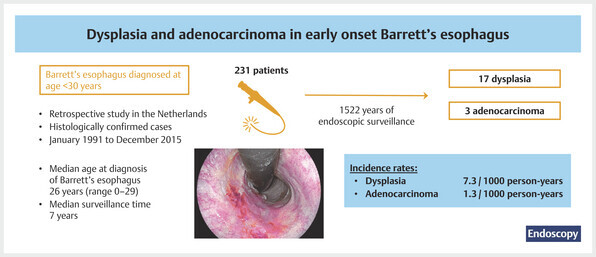

## Introduction

Barrett’s esophagus (BE) is generally defined as a condition in which metaplastic intestinal epithelium with a minimum length of 1 cm, which predisposes to cancer, replaces the stratified squamous epithelium that normally lines the distal esophagus 1 2 3 4 5 *.* The incidence of BE in adults in the Netherlands is estimated to be between 3% and 5%, and it is usually diagnosed when patients are aged 50 years or older 6 . Worldwide, the prevalence in the general population was found to be 1.6% in Sweden 7 and 6.8% in the midwestern USA 8 . Epidemiologic data on the incidence of BE at a younger age are lacking worldwide. In this study, early onset BE was defined as a diagnosis of BE before the age of 30 years, this being an arbitrary choice. For this specific group of young BE patients, no scientific data are available on the risk of progression and the lifetime risk of cancer, other than by extrapolating the progression to adenocarcinoma.

Risk factors for the development of BE include advanced age (>50 years of age), male sex, white ethnicity, gastroesophageal reflux disease (GERD), hiatal hernia, elevated body mass index, and a predominant pattern of intra-abdominal distribution of body fat 9 10 11 12 13 14 . The risk of developing esophageal adenocarcinoma in BE has been found to be 6.3/1000 person-years of follow-up 5 15 ; it shows a positive correlation with the length of the metaplastic epithelium 16 . Regular endoscopic surveillance after a diagnosis of nondysplastic BE of more than 1 cm is recommended in order to detect dysplasia and early esophageal adenocarcinoma with the option of cure with organ-sparing endoscopic therapy. European guidelines recommend surveillance every 5 years when nondysplastic BE with a length of 1–3 cm has been diagnosed and every 3 years with a length between 3–10 cm 6 17 . A previous study has demonstrated that a prolonged period with the presence of BE increases the likelihood of developing adenocarcinoma 16 *.* Therefore, young patients especially have a higher risk of developing adenocarcinoma, as their remaining mean life expectancy from their time of diagnosis is 50 years or more 18 .

As there is no structural data collection or registration of cases of early onset BE in the Netherlands, nationwide data are not available. Our aim was to obtain epidemiologic data on the incidence of dysplasia or adenocarcinoma in early onset BE in the Netherlands by collecting data on all histologically confirmed cases over a prolonged period of 25 years. The development of dysplasia or adenocarcinoma in early onset BE is the primary end point of this study.

## Methods

This retrospective nationwide population-based study was reviewed and approved by the institutional Review Board of the Catharina Hospital and the Medical Research Ethics Committees United (MEC-U; trial no. w16.127).

### Data source

The data for this study were obtained from the Dutch National Pathology Registry (PALGA). PALGA is the national network and registry of histo- and cytopathology in the Netherlands. The database was established in 1971 by the PALGA foundation and reached full national coverage from 1991 onwards. Pathology reports are received on a daily basis and are automatically transposed into standardized excerpts containing pseudonymized patient data, a pathologist’s conclusion, and a PALGA diagnosis based upon the Dutch version of the Systemized Nomenclature of Medicine (SNOMED) 19 *.* PALGA works under strict privacy conditions and the provision of potentially identifiable personal information is strictly governed. All positive hits of the PALGA search are numbered consecutively and the dataset provided for research is sanitized to protect the privacy of patients.

### Search strategy

Nationwide histologically confirmed incidence data on early onset BE were collected with a PALGA search containing the following terms: intestinal metaplasia in the esophagus, or BE. The initial selection included excerpts generated for patients who were younger than 30 years of age between January 1, 1991 and December 31, 2015.

### Definition

Between 1991 and 2015, the nomenclature of BE has changed several times and there was no strict division in the metaplastic subtypes: intestinal-, cardiac-, gastric-, and fundic-type metaplasia were all called Barrett’s metaplasia. Nowadays, BE is defined as the presence of intestinal metaplasia visualized during endoscopic examination with histologically proven metaplastic columnar epithelium that contains prominent goblet cells in the esophagus. There is evidence that only metaplastic intestinal epithelium predisposes to cancer and only those patients are submitted into strict surveillance protocols based on (inter)national guidelines 6 17 20 .

### Data selection

The results retrieved from the PALGA database contained a short summary of the histology report (microscopy and conclusion). The excerpt evaluation was performed by a trained investigator (I.N.) after agreement on the diagnostic criteria with an experienced pathologist (C.H.). We requested the complete histology report in cases where the subtype of BE metaplasia was unclear from the microscopy and histology report. If there was histologically proven metaplastic columnar epithelium in the esophagus, we requested the endoscopy report. The process of acquiring endoscopy reports was facilitated by PALGA and the local pathology laboratory in combination with the treating endoscopist/gastroenterologist from the specific hospital. When there was a suspicion of BE visualized in the esophagus during the endoscopic examination, in combination with targeted biopsies of the visualized BE and a concordant histologic diagnosis of intestinal metaplasia, the patient was included in the study.

### End points

The primary end point was the incidence of dysplasia or adenocarcinoma in early onset BE. The secondary end points included: sex ratio, age at diagnosis, number of surveillance endoscopies, surveillance time in years, time between diagnosis of early onset BE and diagnosis of dysplasia (in years).

### Data abstraction and analyses

For each patient, we included data on sex, age at diagnosis of early onset BE, date of diagnosis, presence of dysplasia or adenocarcinoma at the time of diagnosis (defined as dysplasia or adenocarcinoma at baseline), date of surveillance endoscopies, and histology at surveillance. Baseline was defined as the date of the first biopsy to diagnose early onset BE and considered as the start of the surveillance period. The surveillance time was defined as the time in years between baseline and the end of follow-up on September 1, 2022. A diagnosis of dysplasia or adenocarcinoma within the first 6 months of follow-up was considered to have possibly been missed during index endoscopy. Progression to dysplasia or adenocarcinoma was categorized as prevalent dysplasia/adenocarcinoma when the progression occurred within the first 6 months of follow-up, or incident dysplasia/adenocarcinoma when the progression occurred after the first 6 months of follow-up.

The primary outcome consisted of the incidence rates of the development of dysplasia or adenocarcinoma in early onset BE. The development of dysplasia or adenocarcinoma was defined as any patient found to have dysplasia or adenocarcinoma over the course of the study after the first 6 months of follow-up.

The incidence rate (per 1000 person-years) of dysplasia was calculated by dividing the total number of patients who developed dysplasia by the total number of observed person-years of surveillance. The incidence rate of adenocarcinoma was similarly constructed.

Descriptive statistics were performed using SPSS (IBM SPSS Statistics, version 28.0.023). The mean (SD) was calculated for normally distributed continuous variables and the median and interquartile range (IQR) for continuous variables that were not normally distributed.

## Results

**Fig. 1:**
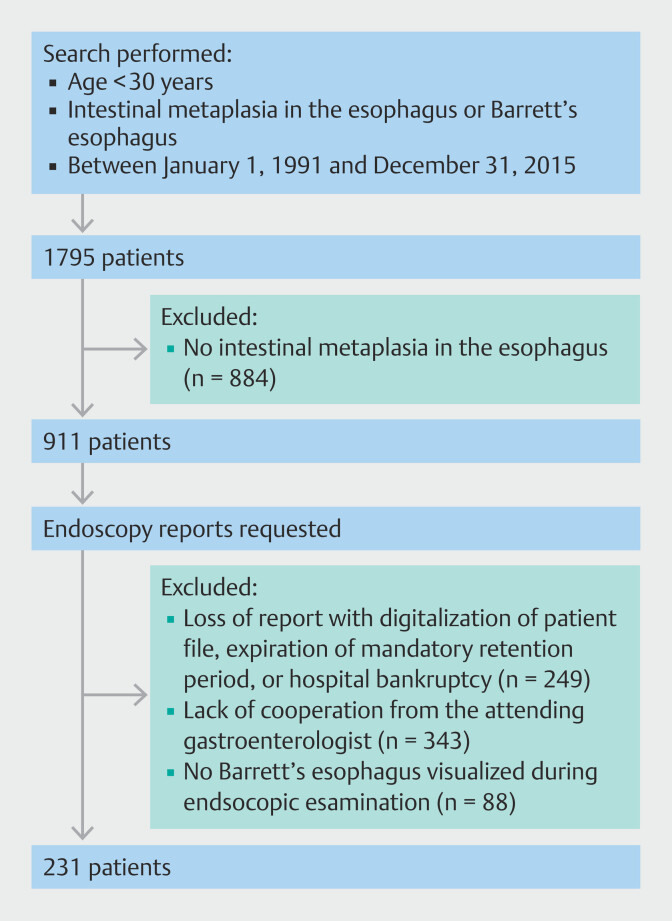
Study flowchart showing the inclusion and exclusion of patients to produce the final cohort of 231 patients with endoscopically and histologically confirmed early onset Barrett’s esophagus.

Between January 1991 and December 2015, 1795 patients were identified ( Fig. 1 ). After review of the pathology excerpts or requested additional pathology reports, 884 patients without intestinal metaplasia in the esophagus were excluded. Endoscopy reports were requested for a total of 911 eligible patients. Of these 911 eligible patients, endoscopy reports were not available for 249 owing to loss of the report with digitization of the patient file, expiration of the mandatory retention period, or closure of the hospital (for financial reasons). For 343 patients, there was no cooperation from the attending gastroenterologist and therefore no availability of the endoscopy reports. Finally, we examined endoscopy reports of 319 patients. Biopsies from intestinal metaplasia visualized during endoscopic examination had been taken for 231 patients, whereas 88 patients had no visualized early onset BE during endoscopic examination. Therefore, the final study population consisted of 231 early onset patients with histologically confirmed BE in biopsies taken from intestinal metaplasia visualized in the esophagus during endoscopic examination.

**Fig. 2:**
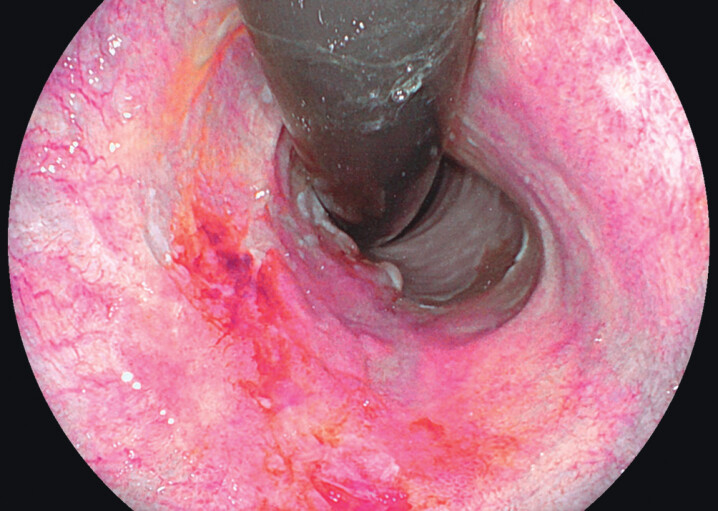
Endoscopic view of early onset Barrett’s esophagus with early adenocarcinoma (Prague C3M5 with a Paris type IIA/IIB lesion), taken with a Fujifilm gastroscope (HD EG760) and the linked-color imaging (LCI).

### Patient characteristics

The median age of the study cohort at time of their diagnosis of early onset BE was 26 years (range 0–29 years), with the majority being male (84%). Of the 231 patients, 172 (74.5%) received at least one surveillance endoscopy with a biopsy procedure; the median number of surveillance endoscopies was 3 (range 1–22). The median surveillance time was 7 years (range 0–29 years). We analyzed a total of 1522 years of early onset BE surveillance in 172 patients. At baseline 97.0% of the patients had intestinal metaplasia without dysplasia. Low grade dysplasia (LGD) was detected in five patients (2.2%), high grade dysplasia (HGD) in one patient (0.4%), and one patient was diagnosed with adenocarcinoma ( Fig. 2 ). The baseline characteristics of the patients are shown in Table 1 .

**Table:** **Table 1** Baseline characteristics and surveillance findings of the 231 patients diagnosed with early onset Barrett’s esophagus in the Netherlands between 1991 and 2015.

Characteristic/finding	n (%), unless otherwise stated
Baseline	
Sex, male	194 (84.0)
Age at diagnosis, median (IQR), years	26 (0–29)
Histology at baseline	
No dysplasia	224 (97.0)
Low grade dysplasia	5 (2.2)
High grade dysplasia	1 (0.4)
Adenocarcinoma	1 (0.4)
Surveillance endoscopy	
Yes	172 (74.5)
No	59 (25.5)
During surveillance ^1^	
Number of surveillance endoscopies, median (IQR)	3 (1–22)
Surveillance time, median (IQR), years	7 (0–29)
Worst histology at surveillance	
No dysplasia	153 (89.0)
Low grade dysplasia	14 (8.1)
High grade dysplasia	2 (1.2)
Adenocarcinoma	3 (1.7)
IQR, interquartile range. ^1^ n = 172 (i.e. those patients who underwent at least one surveillance endoscopy after diagnosis of early onset Barrett’s esophagus).	

### Progression to dysplasia

A total of 17 patients were diagnosed with dysplasia. In six patients, the dysplasia was classified as prevalent (5 LGD and 1 HGD). The median age at the time of dysplasia diagnosis for these patients was 25 years (range 0–27 years), with the majority (5 patients) being male. Five patients received at least one surveillance endoscopy and the median surveillance time after diagnosing dysplasia was 8 years (range 0–16 years).

In 11 patients, the progression to dysplasia was classified as incident (11 LGD) ( Table 2 ). The median age at the time of dysplasia diagnosis in these patients was 28 years (range 13–29 years), with the majority (10 patients) being male; all had intestinal metaplasia at baseline and were diagnosed with dysplasia during surveillance (10 LGD and 1 HGD). The median surveillance time between the diagnosis of early onset BE and the diagnosis of dysplasia was 5 years (range 0–16 years), with patients undergoing a median of three surveillance endoscopies (range 1–6). The median surveillance time between the last screening endoscopy that showed intestinal metaplasia without dysplasia and the endoscopy in which dysplasia was detected was 25 months (range 5–54 months). The median time from the diagnosis of LGD to the subsequent surveillance was found to be 11 months (range 2–35). The median surveillance time after the diagnosis of dysplasia was 13 years (range 1–24).

**Table:** **Table 2** Characteristics of the patients with incident dysplasia in early onset Barrett’s esophagus (BE) in the Netherlands between 1991 and 2015.

Characteristic/finding	n (%), unless otherwise specified
Dysplasia	11 (4.7 ^1^ )
Sex, male	10 (90.9)
Age at diagnosis of dysplasia, median (IQR), years	28 (13–29)
Worst histology	
Low grade dysplasia	10 (4.3 ^1^ )
High grade dysplasia	1 (0.4 ^1^ )
Total number of surveillance endoscopies, median (IQR)	7 (3–22)
Total surveillance time, median (IQR), years	16 (6–29)
Time between diagnosis of early onset BE and diagnosis of dysplasia, median (IQR), years	5 (1–16)
IQR, interquartile range. ^1^ Percentage of total cohort size at baseline.	

The incidence rate of dysplasia in early onset BE was found to be 7.3 (95%CI 4.5–17.5) per 1000 person-years.

### Progression to adenocarcinoma

Three patients were diagnosed with adenocarcinoma. A 28-year-old woman was diagnosed with HGD at baseline and after 3 months was diagnosed with adenocarcinoma. This was considered as have possibly been missed during the index endoscopy and thereby diagnosed as prevalent adenocarcinoma. After the diagnosis of adenocarcinoma, the patient underwent endoscopic resection and the surveillance time after endoscopic resection was 58 months.

Two patients developed adenocarcinoma during surveillance. A 35-year-old man was diagnosed after 9 years of surveillance from his diagnosis of early onset BE. The time between his last screening endoscopy without adenocarcinoma and the endoscopy during which adenocarcinoma was detected was 38 months. After being diagnosed with adenocarcinoma, the patient underwent endoscopic resection, with a surveillance time after endoscopic resection of 7 months. A 36-year-old man was also diagnosed after 9 years of surveillance from his diagnosis of early onset BE. The time between his last screening endoscopy without adenocarcinoma and the endoscopy during which adenocarcinoma was detected was 14 months. After his diagnosis of adenocarcinoma, the patient underwent endoscopic resection, with a surveillance time after endoscopic resection of 9 months. This corresponded to an incidence rate of adenocarcinoma in early onset BE of 1.3 (95%CI 0.8–4.8) per 1000 person-years.

## Discussion

The aim of this retrospective population-based study was to investigate data on the incidence of dysplasia and adenocarcinoma in early onset BE in the Netherlands. To the best of our knowledge, this is the first study to ever report on the incidence of dysplasia and adenocarcinoma in early onset BE.

Our results showed that, before 2015, at least 231 patients were diagnosed with early onset BE, of whom 17 patients (7.4%) were diagnosed with dysplasia and three (1.3%) with adenocarcinoma, either at baseline or during surveillance. Surveillance data were available up until September 1, 2022, which resulted in a prolonged period of 29 years of surveillance for those patients who were included shortly after initiation of the PALGA database. We analyzed a total of 1522 years of early onset BE surveillance in 172 patients. The incidence rates of dysplasia in early onset BE was established as being 7.3 (95%CI 4.5–17.5) per 1000 person-years and of adenocarcinoma as being 1.3 (95%CI 0.8–4.8) per 1000 person-years.

The incidence rate of adenocarcinoma in early onset BE identified in our study is comparable with the 2.5 per 1000 person-years mentioned in a large community-based US cohort of BE patients 10 ; however, the age at diagnosis was higher in the US study population (mean age 61 years). In patients diagnosed with BE, it is evident that a study population with a higher mean age, by definition, has a lower remaining life expectancy, owing to the various factors that influence overall mortality. We hypothesize an increase in age of diagnosis correlates with a larger time difference between onset and diagnosis of BE. This might partially explain why the expected lifetime for a patient after a diagnosis of BE is made at young age is longer than that for those diagnosed at a higher mean age.

Klaver et al. assessed the risk for progression to HGD or adenocarcinoma in a Dutch prospective cohort of BE patients 21 . They found progression to HGD or adenocarcinoma in 8.7% of patients, with a mean age of 57 years (SD 11.5 years) at the time of dysplasia diagnosis. Roumans et al. evaluated sex differences in the progression to HGD or adenocarcinoma in a multicenter prospective cohort study 22 . They found progression to HGD or adenocarcinoma in 7% of patients, with a median age of 60 years (IQR 52–69 years) in men. In our study, 2/231 patients (0.9%) progressed to adenocarcinoma after 9 years surveillance. An important factor for neoplastic progression identified in the past was higher age at diagnosis 11 . The study population of our study was more than 25 years younger, which might also explain the lower percentage rates of progression that were found.

Based on the available data, we assume that the number of patients with early onset BE would have been higher if we had been able to obtain all of the requested pathology and endoscopy reports in the Netherlands. Endoscopy reports of 592 patients were not available because of loss of the report or lack of cooperation from the attending gastroenterologist. Of the 319 endoscopy reports received, 231 patients (74.2%) were diagnosed with early onset BE. If we assume this percentage were to be stable across the population, 439 of the 592 excluded patients might have been diagnosed with early onset BE. A total of 670 patients with early onset BE could have been diagnosed in 24 years, which would mean 28 new cases per year.

Data from the Dutch cancer registry showed, between 1991 and 2021, 31 patients were diagnosed with esophageal adenocarcinoma while aged under 30 years (average of about 1 per year) 23 . If it is assumed that esophageal adenocarcinoma below the age of 30 years is nearly always associated with early onset BE, these data suggest that early onset BE is underdiagnosed and under-reported. Some of the 31 patients diagnosed with esophageal adenocarcinoma might have been diagnosed with early onset BE after 2015 and they would therefore not have been included in our study population. We detected esophageal adenocarcinoma in three patients within our study population. More patients with esophageal adenocarcinoma were most likely not identified because of one of the following factors. First, the diagnosis of early onset BE could not be endoscopically confirmed: five patients with histologically proven metaplastic columnar epithelium in the esophagus and development of adenocarcinoma in surveillance were excluded because endoscopy reports were not available. Second, patients were not diagnosed with early onset BE, because the diagnosis was made at the index endoscopy and biopsies were taken only from the carcinoma itself.

We suggest that our missing data during data selection are missing completely at random because we expect that patients with young onset BE are evenly distributed in the Netherlands. The Netherlands is a small country with a homogeneous population distribution. We therefore would not expect there to be any factors affecting the distribution of patients with young onset BE. All involved local pathology laboratories and hospitals were approached in the same way and we did not make any distinction ourselves. The hospitals completed their own search and reported back if data were no longer available or when they no longer wished to cooperate. We expect that the missing reports do not depend on either the observed and unobserved data. We therefore expect that our calculated incidence rates of dysplasia and adenocarcinoma in early onset BE reflect the incidence rates in the Netherlands.

Our data show that the median surveillance time between the last screening endoscopy with intestinal metaplasia without dysplasia and the endoscopy during which dysplasia was detected was 24 months. This interval is considered to be adequate, as the guidelines suggest an interval of 3 or 5 years, depending on the length of the BE 17 .

The time between the diagnosis of LGD and the subsequent surveillance endoscopy was found to be 11 months. Current guidelines advise reviewing biopsies that were taken during an endoscopy where there was LGD 6 17 . If these biopsies confirm the diagnosis of LGD, it is suggested to schedule a surveillance endoscopy within 6 months. Unfortunately, data on whether the LGD diagnosis was confirmed on review of these biopsies are lacking; however, given the suggested surveillance interval of 6 months, a period of 11 months between diagnosis and surveillance as found in the current study is not in line with the advice in current guidelines.

This study has several limitations that should be addressed. The most important limitation of this study is the missing data in terms of endoscopy reports that could not be retrieved for various reasons (i.e. lost during digitization, or lack of cooperation from the medical specialist), which resulted in 343 missing endoscopy reports. We suggest that our incomplete data are missing completely at random. Nevertheless this is a unique study reporting on the incidence rate of progression to dysplasia or adenocarcinoma in early onset BE. The incidence of early onset BE has not been studied before. A second limitation is that potentially interesting information, including the length of the BE at baseline, the considerations of the treating gastroenterologist on (the timing of) the surveillance gastroscopies, whether biopsies were taken in line with the Seattle protocol, and information on review of the diagnosis after a diagnosis of LGD, was not available as it is not routinely collected by PALGA, nor reported in the endoscopy report. These parameters should be addressed in further studies.

From our perspective, the findings of the current study indicate the relevance of studying the incidence of early onset BE and investing in future research. The results provide us with the opportunity to discuss whether guidelines written for patients with adult onset of BE should also be followed for patients with early onset BE. Are early onset BE patients, when diagnosed below 18 years of age, transferred from pediatricians to gastroenterologists? Currently, we have insufficient information to answer these questions. For future studies, it will be important to prospectively gather additional information that could provide more data on the development of dysplasia or adenocarcinoma in these young adults. Such studies are expected to give more insight into the important risk factors for progression among patients with early onset BE. These factors would be important in making a more accurate individual risk assessment for progression to be able to select those patients with the highest risk of developing dysplasia or adenocarcinoma. It could therefore be valuable to discuss whether all patients with early onset BE need to be referred to a BE expert center for surveillance. Furthermore, it could be interesting to analyze the cost-effectiveness of treatment for intestinal metaplasia in the esophagus with radiofrequency ablation to cure the early onset BE and protect patients from the presumed high lifetime risk for progression to dysplasia or adenocarcinoma. Subsequently, it could be discussed whether it is cost-effective to treat all patients with early onset BE, or only those patients with the highest risk of progression to dysplasia or adenocarcinoma.

In conclusion, this is the first descriptive study with a population-based design to report on the incidence rates of progression to dysplasia and adenocarcinoma in early onset BE worldwide. Between 1991 and 2015, 231 patients were diagnosed with early onset BE in the Netherlands, of whom 17 patients (7.4%) were diagnosed with dysplasia and three (1.3%) with adenocarcinoma during a total of 1522 years of BE surveillance. The incidence rate of dysplasia in early onset BE was 7.3 (95%CI 4.5–17.5) per 1000 person-years and for adenocarcinoma was 1.3 (95%CI 0.8–4.8) per 1000 person-years.

## Supplementary Material

Supplementary Material Zusatzmaterial
