# Supplementary material for: Exploring the incidence of dysplasia or adenocarcinoma in early onset Barrett’s esophagus
Source: Endoscopy. 2024 Sep 23;56(12):906–12. doi: 10.1055/a-2386-7843 (PMC11716541; doi:10.1055/a-2386-7843)
Supplement: Supplementary file 1 — Supplementary Material [file 10-1055-a-2386-7843_23871047.pdf]

Supplementary material

Acknowledgments

We would like to thank the pathologists from the following laboratories for their cooperation with this study:

Admiraal de Ruyter Hospital, Goes, the Netherlands

Alrijen Hospital, Leiden, the Netherlands

Amphia Hospital, Breda, the Netherlands

Amsterdam University Medical Centers, location AMC, the Netherlands

Amsterdam University Medical Centers, location VU, the Netherlands

Antonius Hospital, Nieuwegein, the Netherlands

Canisius Wilhelmina Hospital, Nijmegen, the Netherlands

Diakonessenhuis, Utrecht, the Netherlands

Erasmus MC, Rotterdam, the Netherlands

Gelre Hospital, Apeldoorn, the Netherlands

Groene Hart Hospital, Gouda, the Netherlands

Haga Teaching Hospital, den Haag, the Netherlands

Isala Clinics, Zwolle, the Netherlands

Jeroen Bosch Hospital, den Bosch, the Netherlands

Laboratory clinical pathology middle Brabant, the Netherlands

Laurentius Hospital, Roermond, the Netherlands

Leiden University Medical Center, Leiden, the Netherlands

Maasstad Hospital, Rotterdam, the Netherlands

Martini Hospital, Groningen, the Netherlands

MC Alkmaar, Alkmaar, the Netherlands

MC Haaglanden, den Haag, the Netherlands

OLVG, Amsterdam, the Netherlands

Pathology and cytology laboratory West-Brabant, the Netherlands

Pathology laboratory Dordrecht, Dordrecht, the Netherlands
Radboud University Medical Center, Nijmegen, the Netherlands

Rijnstate Hospital, Arnhem, the Netherlands

Pathan laboratory, Rotterdam, the Netherlands

RPCL, Amersfoort, the Netherlands

S.S.D.Z Reinier de Graaf, Delft, the Netherlands

 | © 2024. Thieme. All rights reserved.

Supplementary material

Spaarne Gasthuis, Haarlem, the Netherlands

Stichting Labaratorium Pathologie Oost Nederland, the Netherlands

Stichting PAMM, the Netherlands

Tergooiziekenhuizen, the Netherlands

Universitair Medical Centre Groningen, Groningen, the Netherlands

Universitair Medical Centre Maastricht, Maastricht, the Netherlands

Universitair Medical Centre Utrecht, Utrecht, the Netherlands

VieCuri Medical Center, Venlo, the Netherlands

Zuyderland MC, Heerlen, the Netherlands

Zuyderland MC, Sittard, the Netherlands

We would like to kindly acknowledge the gastroenterologists from the following hospitals for their contribution to this study:

Albert Sweitzner Hospital, Dordrecht, the Netherlands

Amstelland Hospital, Amstelveen, the Netherlands

Amsterdam University Medical Centers, location AMC, the Netherlands

Amphia Hospital, Breda, the Netherlands

Antonius Hospital, Nieuwegein, the Netherlands

Beatrix Hospital, Gorinchem, the Netherlands

Diakonessenhuis, Utrecht, the Netherlands

Elisabeth Tweesteden Hospital, Tilburg, the Netherlands

Elkerliek hospital, Helmond, the Netherlands

Erasmus MC, Rotterdam, the Netherlands

Gelderse Vallei, Ede, the Netherlands

Gelre Hospital, Apeldoorn, the Netherlands

Groene Hart Hospital, Gouda, the Netherlands

Haga Teaching Hospital, den Haag, the Netherlands
Ikazia, Rotterdam, the Netherlands

Isala Clinics, Zwolle, the Netherlands

Jeroen Bosch Hospital, den Bosch, the Netherlands

Laurentius Hospital, Roermond, the Netherlands

Leiden University Medical Center, Leiden, the Netherlands

© 2024. Thieme. All rights reserved.

Supplementary material

Maasstad Hospital, Rotterdam, the Netherlands

Maxima MC, Veldhoven, the Netherlands

MC Haaglanden, den Haag, the Netherlands

MC Leeuwarden, Leeuwarden, the Netherlands

Nij Smellinghe Hospital, Drachten, the Netherlands

Radboud University Medical Center, Nijmegen, the Netherlands

Rijnstate Hospital, Arnhem, the Netherlands

Reinier de Graaf Hospital, Delft, the Netherlands

Slingeland Hospital, Doetinchem, the Netherlands

Spaarne Gasthuis, Haarlem, the Netherlands

St. Anna Hospital, Geldrop, the Netherlands

St. Jans Gasthuis, Weert, the Netherlands

Universitair Medical Centre Maastricht, Maastricht, the Netherlands

Universitair Medical Centre Utrecht, Utrecht, the Netherlands

ZGT, Almelo, the Netherlands

Zuyderland MC, Sittard, the Netherlands

Zuyderland MC, Heerlen, the Netherlands

 | © 2024. Thieme. All rights reserved.
